# Phosphoproteomic analysis sheds light on intracellular signaling cascades triggered by Formyl-Peptide Receptor 2

**DOI:** 10.1038/s41598-019-54502-6

**Published:** 2019-11-29

**Authors:** Fabio Cattaneo, Rosita Russo, Martina Castaldo, Angela Chambery, Cristiana Zollo, Gabriella Esposito, Paolo Vincenzo Pedone, Rosario Ammendola

**Affiliations:** 1 Department of Molecular Medicine and Medical Biotechnology, University of Naples Federico II, 80131 Naples, Italy; 2 Department of Environmental, Biological and Pharmaceutical Sciences and Technologies, University of Campania “Luigi Vanvitelli”, 81100 Caserta, Italy

**Keywords:** Biochemistry, Cell biology

## Abstract

Formyl peptide receptors (FPRs) belong to the family of seven transmembrane Gi-protein coupled receptors (GPCR). FPR2 is considered the most promiscuous member of this family since it recognizes a wide variety of ligands. It plays a crucial role in several physio-pathological processes and different studies highlighted the correlation between its expression and the higher propensity to invasion and metastasis of some cancers. FPR2 stimulation by its synthetic agonist WKYMVm triggers multiple phosphorylations of intracellular signaling molecules, such as ERKs, PKC, PKB, p38MAPK, PI3K, PLC, and of non-signaling proteins, such as p47^phox^ and p67^phox^ which are involved in NADPH oxidase-dependent ROS generation. Biological effects of FPR2 stimulation include intracellular Ca^2+^ mobilization, cellular proliferation and migration, and wound healing. A systematic analysis of the phosphoproteome in FPR2-stimulated cells has not been yet reported. Herein, we describe a large-scale phosphoproteomic study in WKYMVm-stimulated CaLu-6 cells. By using high resolution MS/MS we identified 290 differentially phosphorylated proteins and 53 unique phosphopeptides mapping on 40 proteins. Phosphorylations on five selected phospho-proteins were further validated by western blotting, confirming their dependence on FPR2 stimulation. Interconnection between some of the signalling readout identified was also evaluated. Furthermore, we show that FPR2 stimulation with two anti-inflammatory agonists induces the phosphorylation of selected differentially phosphorylated proteins, suggesting their role in the resolution of inflammation. These data provide a promising resource for further studies on new signaling networks triggered by FPR2 and on novel molecular drug targets for human diseases.

## Introduction

Formyl peptides receptors (FPR1, FPR2, FPR3) are members of the GPCR family coupled to inhibitory G (Gi) proteins sensitive to pertussis toxin (PTX)^1,2^. “FPR2 binds efficiently the synthetic peptide” WKYMVm (W peptide) and is the most promiscuous member of the FPRs family, since peptides and lipids of different origin have been identified as FPR2 agonists^3–5^. FPR2 is highly expressed in cells of the immune system but it is also functionally expressed on cellular membranes of epithelial, neuronal and endothelial cells^1^. FPR2 was also detected onto nuclear membranes of anaplastic lung cancer CaLu-6 and human gastric adenocarcinoma AGS cells, where triggers intranuclear signaling cascades crucially involved in gene expression^6^. Furthermore, different studies highlighted the correlation between FPRs expression or function and inflammatory conditions, including inflammatory disorders, cancer, infections and other diseases^7–9^.

FPR2 stimulation induces multiple phosphorylations of several signalling proteins, which modulate critical intracellular functions such as cell growth, proliferation, intracellular communication, migration, differentiation, apoptosis, and survival^5,10^. For instance, the interaction of W peptide with its receptor elicits the activation of phospholipase A2, phospholipase C, and phospholipase D (PLA2, PLC, PLD)^11,12^, the stimulation of phosphatidylinositol-3-kinase (PI3-K)/protein kinase B (PKB or Akt) pathway^13^, and the activation of protein kinase C (PKC), p38MAPK, extracellular response kinases 1 and 2 (ERK1/2) and c-Src^14,15^. Some of FPR2-dependent phosphorylated molecules are non-signaling proteins, such as the cytosolic subunits p47^phox^ and p67^phox^ of NADPH oxidase complex^16^ whose phosphorylation requires FPR2-dependent activation of ERKs, PKCα, and PKCδ^17^. Activated NADPH oxidase complex generates reactive oxygen species (ROS), which play a crucial role in tyrosine kinase receptors (TKRs) transactivation in several cell lines^18^. In CaLu-6 cell line, FPR2 stimulation by W peptide induces Epidermal Growth Factor Receptor (EGFR), p47^phox^ and c-Src phosphorylation. Phosphotyrosine residues of EGFR provide docking sites for recruitment, phosphorylation, and triggering of Signal Transducer and Activator of Transcription 3 (STAT3) transcriptional factor^16^. In human prostate epithelial cell line PNT1A, FPR2 activation induces the phosphorylation of Y1313, Y1349 and Y1356 residues of Hepatocyte Growth Factor Receptor (c-Met) and triggers STAT3, PLC-γ1/PKCα and PI3K/Akt pathways^19^. Furthermore, N-fMLP stimulation of FPR1 induces the phosphorylation of cytosolic Y951, Y996, and Y1175 residues of Vascular Endothelial Growth Factor Receptor 2 (VEGFR2), which constitute the anchoring sites for signaling molecules. These, in turn, activate PI3K/Akt and PLC-γ1/PKC intracellular pathways^20^.

Phosphorylation represents the most frequent reversible covalent modification of proteins observed in eukaryotic cells. About 2–5% of the human genome codifies for kinases and phosphatases^21^ and about 30% of the total content of proteins in eukaryotic cells is phosphorylated at any given time^22^. However, in eukaryotic cells the overall level of tyrosine and threonine phosphorylated residues in proteins is very low compared to the level of phosphoserine^23^.

Characterization of the phosphorylation status of cells plays a key role in the study of intracellular signaling cascades, in the understanding of the molecular mechanisms responsible of human disorders and, in turn, for the appropriate pharmacological approach^24^.

Although intracellular signaling pathways triggered by W peptide in FPR2-expressing cells are widely investigated, a complete analysis of the phosphoproteome is not currently available. Herein, we apply a phosphoproteomics approach to investigate phosphorylated proteins in WKYMVm-stimulated CaLu-6 cells. We identified 290 phosphoproteins and 53 unique phosphopeptides mapping on 40 proteins and we prove that selective phosphorylation on Ser82 of Heat Shock Protein-27 (HSP-27), Ser139 of Mini-Chromosome Maintenance protein 2 (MCM2), Ser339 of Oxidative-Stress-Responsive kinase 1 (OSR1), Ser608 of Retinoblastoma (Rb), and Ser170 of Myristolated Alanine-Rich C-Kinase Substrate (MARCKS), specifically depends on FPR2 stimulation by WKYMVm. The interconnection between some of the signalling readouts identified was demonstrated by using specific signalling inhibitors. Furthermore, we show that FPR2 stimulation with Annexin A1 (ANXA1) or Lipoxin A4 (LXA4), two anti-inflammatory FPR2 agonists, induces the phosphorylation of the selected differentially phosphorylated proteins.

Our overall data demonstrate that network-based global phospho-proteomic analysis can contribute to the identification of new intracellular pathways elicited by FPR2 which, in turn, could lead to the identification of new targeting strategies for FPR2-dependent human diseases.

## Methods

### Cell culture and reagents

CaLu-6 cell line was obtained from American Type Culture Collection (ATCC, Manassas, VA, USA). Cells were grown in Dulbecco’s modified Eagle’s medium (DMEM) (Thermo Fisher Scientific, Monza, Italy) supplemented with 10% foetal bovine serum (FBS) (Invitrogen). Once reached 80% confluence, cells were serum starved for 24 hours, and stimulated for 5 minutes with WKYMVm (Primm, Milan, Italy) at the final concentration of 10 μM, or with Lipoxin A4 (Santa Cruz, CA, USA) at the final concentration of 1 μM or with Annexin A1 (Abcam) at the final concentration of 10 nM. In other experiments, serum-deprived cells were preincubated for 16 hours with PTX (Sigma) at a final concentration of 100 ng/mL, or with 10 μM WRWWWW (WRW4) (Primm, Milan, Italy) for 15 minutes, or with 50 μM PD098059 (Sigma) for 90 minutes, or with 5 Rottlerin μM (Sigma) for 1 hour before the stimulation with W peptide. SDS-PAGE reagents were purchased from Bio-Rad (Hercules, CA, USA). Anti-phosphoHSP-27(S82), anti-phosphoMCM2(S139) and anti-phosphoRb(S608) were from Cell Signalling Technology (Denvers, MA, USA). Anti-phosphoOSR1(S339) was purchased from Signalway Antibody (Baltimore, MD, USA) and anti-phosphoMARCKS(S170) were from GeneTex (Irvine, CA, USA). Anti-tubulin, anti-cyclin D, anti-cyclin E, anti-rabbit and anti-mouse antibodies were purchased from Santa Cruz Biotechnology (Santa Cruz, CA, USA). Protein A–horseradish peroxidase was from Thermo Scientific (Little Chalfont, Buckinghamshire, UK).

### Phospho-proteins enrichment

The enrichment of phosphorylated proteins was performed by a phosphoprotein purification kit (Qiagen, Hilden, Germany), accordingly to the manufacturer’s instructions. Briefly, whole lysates were obtained from 24-hours serum starved CaLu-6 cells stimulated or not with W peptide for 5 minutes. Cellular proteins were incubated with the appropriate amount of Phosphoprotein Lysis Buffer for 30 minutes at 4 °C and gently vortexed every 10 minutes. Lysates were centrifuged at 10000xg for 30 minutes at 4 °C and protein concentration was determined by using a Bio-Rad protein assay (Biorad, Hercules, CA, USA). Lysates (2.5 mg) were loaded onto Phosphoprotein Purification Column to allow the binding of phosphorylated proteins and the column was washed with the appropriate buffer as described in the manufacturer’s instructions. Finally, phosphoproteins were eluted by adding to the column 500 µL of Phosphoprotein Elution Buffer. This step was repeated four times and the concentration of the four enriched phosphoprotein fractions was determined. To concentrate samples, an ultrafiltration step was performed by centrifuging the eluted fractions, at 10000xg for 10 min.

### Tryptic digestion and sample preparation for MS/MS analyses

Chemicals, tosyl phenylalanyl chloromethyl ketone (TPCK)-treated trypsin were from Sigma Chemical Co. (Milan, Italy). Acetonitrile (CH_3_CN), formic acid (FA) and water LC-MS grade were from Fisher Scientific Italia (Rodano, Milan, Italy).

Aliquots of enriched phosphoprotein samples (~50 µg) from 24-hours serum starved CaLu-6 cells, treated or not with W peptide for 5 minutes, were precipitated by adding pre-chilled acetone (six volumes) for 16 h at −20 °C. Following centrifugation for 10 min at 8,000 × g at 4 °C, protein pellets were resuspended in 100 µL of 50 mM NH_4_HCO_3_ pH 8.2. Samples were then subjected to disulphide reduction with 10 mM DTT (1 h at 55 °C) and alkylation with 7.5 mM iodacetamide (20 min at room temperature in the dark). Enzymatic hydrolyses were performed on reduced and alkylated samples by adding TPCK-treated trypsin with an enzyme/substrate (E/S) ratio of 1:100 (w/w) for 3 h and 1:50 for 16 h at 37 °C.

### High-resolution nanoLC-Tandem mass spectrometry

Mass spectrometry analyses of tryptic digests (2 µg) were performed as previously described^25^ on a Q-Exactive Orbitrap mass spectrometer equipped with an EASY-Spray nanoelectrospray ion source (Thermo Fisher Scientific, Bremen, Germany) and coupled to a ThermoScientific Dionex UltiMate 3000RSLC nano system (Thermo Fisher Scientific).

### Data processing

The acquired raw files were analysed with Proteome Discoverer 2.1 software (Thermo Fisher Scientific) using the SEQUEST HT search engine as described^25^. The HCD MS/MS spectra were searched against the Homo sapiens database available in UniprotKB (20,413 reviewed entries, release 2018_01, 31-Jan-2018) assuming trypsin (Full) as digestion enzyme with two allowed number of missed cleavage sites and a minimum peptide length of six residues. The mass tolerances were set to 10 ppm and 0.02 Da for precursor and fragment ions, respectively. Carbamidomethylation (+57.021464 Da) of cysteine was set as static modification. Phosphorylation of serine, threonine, and tyrosine (+79.966 Da) and acetylation of lysine (+42.011 Da) were set as dynamic modifications. False discovery rates (FDRs) for peptide spectral matches (PSMs) were calculated and filtered using the Target Decoy PSM Validator Node in Proteome Discoverer with the following settings: maximum Delta Cn of 0.05, a strict target FDR of 0.01, a relaxed target FDR of 0.05 and validation based on q-value. Localization and best site probability for variable post-translational modifications within peptides were performed with the ptmRS tool, integrated in Proteome Discoverer^26^. The Protein FDR Validator Node in Proteome Discoverer was used to classify protein identifications based on q-value. Proteins with a q-value of 0.01 were classified as high confidence identifications and proteins with a q-value of 0.01–0.05 were classified as medium confidence identifications. The resulting protein table was then filtered based on the presence of phosphorylated peptides. Phosphopeptide changes following WKYMVm treatment were only considered when modifications were identified in at least two out of three replicate injections in treated sample and not identified in replicate injections of untreated sample.

### Bioinformatic analysis

Interaction network analysis of proteins identified by LC-MS/MS were performed by the FunRich open access software (http://funrich.org/index.html). Molecular functional enrichment of identified proteins based on gene ontology categories was performed by using the Network analyst software (https://www.networkanalyst.ca)^27^.

### Western blotting

Whole lysates were obtained as previously described^28^. CaLu-6 cells were serum-deprived for 24 hours and then stimulated with 10 μM W peptide for 5 minutes. In other experiments, cells were pretreated with the appropriate amount of specific inhibitors before the stimulation with WKYMVm. Briefly, cells were washed in cold phosphate buffered saline (PBS) and lysed by incubation with RIPA buffer (50 mM Tris–HCl, pH 7.4, 150 mM NaCl, 1% NP-40, 1 mM EDTA, 0.25% sodium deoxycholate, 1 mM NaF, 10 μM Na_3_VO_4_, 1 mM phenylmethylsulfonylfluoride, 10 μg/ml aprotinin, 10 μg/ml pepstatin, 10 μg/ml leupeptin) for 45 min at 4 °C^28^. Bio-Rad protein assay was used to determine proteins concentration (BioRAD, Hercules, CA, USA). Fifty micrograms of whole lysates were resolved on 10% SDS-PAGE and proteins were transferred by blotting onto PVDF membranes. Membranes were incubated with phosphospecific primary antibodies and probed with appropriate horseradish peroxidase-conjugated secondary antibodies. Proteins were detected by autoradiography and bands densitometry was estimated using a Discover Pharmacia scanner equipped with a sun spark classic densitometric workstation^29^. The same filters were reprobed with an anti-tubulin antibody to normalize the amount of loaded proteins.

### Statistical analysis

All the data are representative of three or more independent experiments and are expressed as means ± Standard Error Mean (SEM). Statistical analyses were evaluated by one-way analysis of variance (ANOVA) performed with GraphPad Prism 7 (GraphPad Softweare, San Diego, CA, USA). A p value of less than 0.05 was considered statistically significant.

## Results

### Phosphoproteomic analysis by high-resolution nanoLC−MS/MS

**Figure 1:**
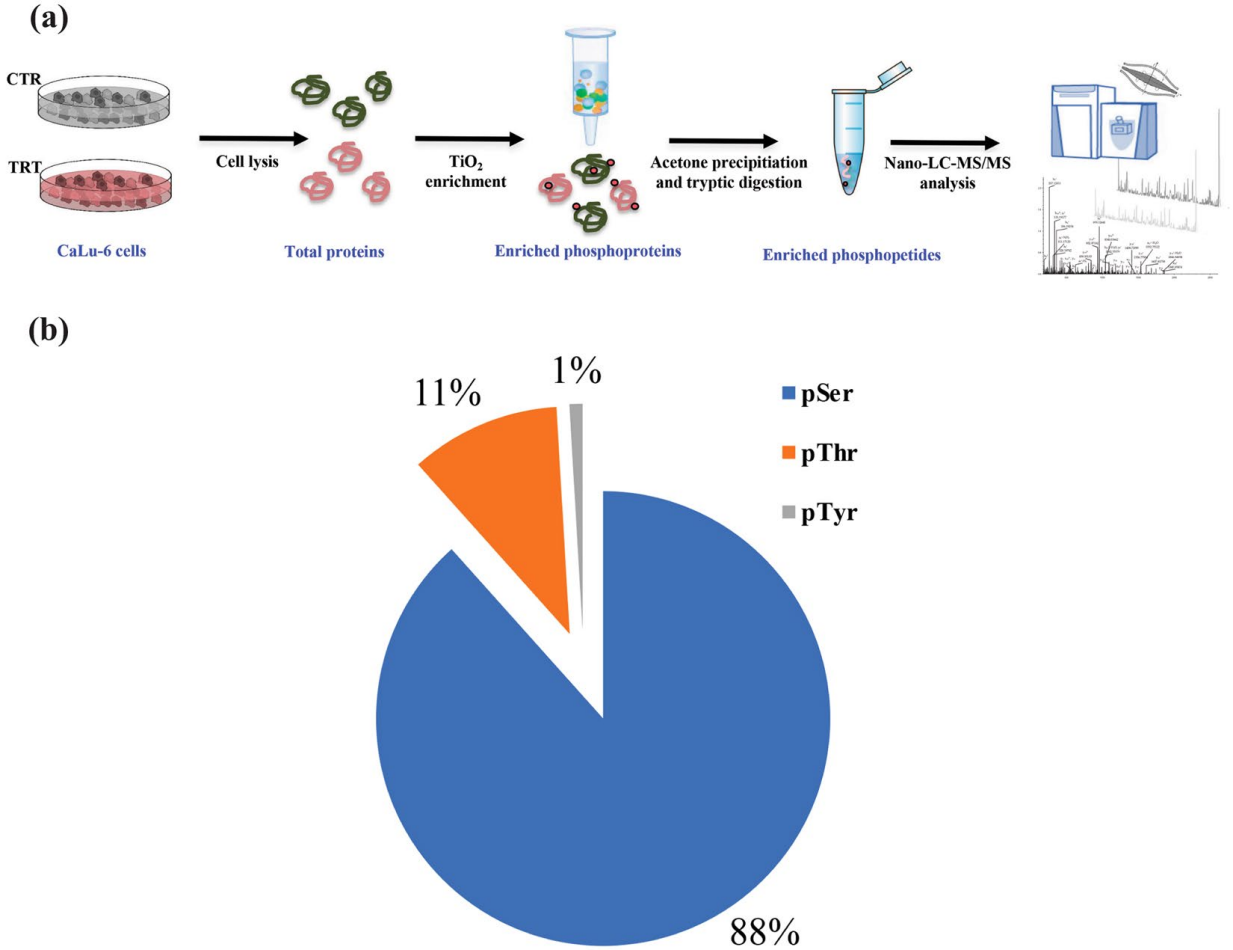
Phosphoproteomic analysis of WKYMVm-stimulated CaLu-6 cells. (**a**) Schematic diagram of the experimental workflow applied for the analysis of CaLu-6 phosphoproteome in response to WKYMVm stimulation by nano-liquid chromatography coupled with high-resolution tandem mass spectrometry. Total proteins were purified in control (CTR) and stimulated (TRT) cells. Phosphoproteins were enriched by TiO_2_ procedure, concentrated by ultrafiltration and digested with tripsin. (**b**) Pie chart of the phosphorylated amino acid distribution.

The global phospho-signaling response triggered by WKYMVm in CaLu-6 cells in comparison to untreated cells was investigated by nano-liquid chromatography coupled with high-resolution tandem mass spectrometry. The schematic representation of the experimental design is reported in Fig. 1a. A high number of peptide groups (i.e. 14723) were used for the identification of 2134 proteins (Supplementary material, Table S1). The phosphoproteomic workflow enabled the identification of 430 phosphorylation sites from 510 phospho-peptides identified in at least one out of the two analyzed conditions (Supplementary material, Table S2A) mapping on 290 proteins (Supplementary material, Table S2B). Localization and best site probability for variable post-translational modifications within peptides were performed with the ptmRS tool of the Proteome Discoverer software that calculates individual probability values for each putatively modified site based on MS/MS data. Analysis of the phosphorylated amino acid distribution showed that 88% of phosphorylated sites were on serine, whereas 11% and 1% were threonine and tyrosine phosphorylation sites, respectively (Fig. 1b). These results are in line with previous literature data reporting that in human cells the overall levels of protein phosphotyrosines and phosphothreonines are lower than those of phosphoserines^23,30,31^.

In order to unravel the biological relevance of phosphorylation events triggered by W peptide stimulation, we focused our attention on a small number of peptides (53 mapping on 40 proteins) that were phosphorylated only in WKYMVm-stimulated cells (Table 1).

**Table 1:** Phosphopeptides only identified in W peptide-stimulated CaLu-6 cells.

Accessions	Description	Peptide Sequence	Positions	ptmRS Probabilities
O60256-1	PRPP synthase-associated protein 2	[R].LGIAVIHGEAQDAESDLVDGRHSPPMVR.[S]	[205–232]	S23: 100
O95425–3	Isoform SV3 of Supervillin	[R].SPSFGDPQLSPEARPR.[CV]	[261–276]	S10: 100
O95747	Serine/threonine-protein kinase OSR1	[R].LHKTEDGGWEWSDDEFDEESEEGK.[A]	[328–351]	S12: 100
P04792	Heat shock protein beta-1	[R].QLSSGVSEIR.[H]	[80–89]	S3: 100
P06400	Retinoblastoma-associated protein	[K].DREGPTDHLESACPLNLPLQNNHTAADMYLSPVRSPK.[K]	[578–614]	S31: 98.09; S35: 100
P06748	Nucleophosmin	[K].MQASIEKGGSLPKVEAK.[F]	[251–267]	S4: 100
P07910-1	Heterogeneous nuclear ribonucleoproteins C1	[K].MESEGGADDSAEEGDLLDDDDNEDRGDDQLELIK.[D]	[238–271]	S10: 100
P16401	Histone H1.5	[K].KATKSPAKPK.[A]	[185–194]	S5: 100
P23588	eukaryotic translation initiation factor 4B	[R].ERHPSWRSEETQER.[E]	[402–415]	S5: 99.37
P29590	Protein PML	[R].SPVIGSEVFLPNSNHVASGAGEAEER.[V]	[530–555]	S1: 100
P29966	Myristoylated alanine-rich C-kinase substrate	[K].SFKLSGFSFKK.[N]	[163–173]	S8: 100
P31943	Heterogeneous nuclear ribonucleoprotein H 1	[K].HTGPNSPDTANDGFVR.[L]	[99–114]	S6: 100
P38159-1	RNA-binding motif protein,X chromosome	[R].DVYLSPRDDGYSTKDSYSSR.[D]	[204–223]	S5: 100
P42167	Lamina-associated polypeptide 2	[R].AKTPVTLK.[Q]	[206–213]	T3: 100
P49006	MARCKS-related protein	[K].KFSFKKPFK.[L]	[91–99]	S3: 100
P49585	choline-phosphate cytidylyltransferase A	[R].HKAAAYDISEDEED.[−]	[354-367]	S9: 100
P51991-1	Heterogeneous nuclear ribonucleoprotein A3	[R].SSGSPYGGGYGSGGGSGGYGSRRF.[−]	[355–378]	S4: 100
Q01518-1	adenylyl cyclase-associated protein 1	[R].SGPKPFSAPKPQTSPSPKR.[A]	[295–313]	T13: 99.99; S14: 50; S16: 50
Q01581	hydroxymethylglutaryl-CoA synthase, cytoplasmic	[R].RPTPNDDTLDEGVGLVHSNIATEHIPSPAK.[K]	[469–498]	S27: 100
Q04637-8	Isoform 8 of eIF-4-gamma 1	[R].EAALPPVSPLKAALSEEELEKK.[S]	[1225–1246]	S8: 100
Q13442	28 kDa heat- and acid-stable phosphoprotein	[K].SLDSDESEDEEDDYQQKRK.[G]	[57–75]	S4: 100; S7: 100
Q15424-3	Isoform 3 of Scaffold attachment factor B1	[R].SVVSFDKVKEPR.[K]	[601–612]	S4: 100
Q8IU81	Interferon regulatory factor 2-binding protein 1	[R].AGGASPAASSTAQPPTQHR.[L]	[449–467]	S5: 100
Q8NC51-1	PAI1 RNA-binding protein 1	[R].GGSGSHNWGTVKDELTESPKYIQK.[Q]	[217–240]	S18: 96.8
Q8WUD4	Coiled-coil domain-containing protein 12	[R].LKGQEDSLASAVDAATEQKTCDSD.[−]	[143–166]	S23: 100
Q8WW12	PEST proteolytic signal-containing nuclear protein	[K].TLSVAAAFNEDEDSEPEEMPPEAKMR.[M]	[106–131]	S14: 100
Q8WY36	HMG box transcription factor BBX	[R].TADGRVSPAGGTLDDKPKEQLQR.[S]	[838–860]	S7: 99.99
Q92538	BFA-resistant GEF 1	[R].GYTSDSEVYTDHGRPGK.[I]	[1315–1331]	S4: 99.46
Q96A00	Protein phosphatase 1 regulatory subunit 14 A	[R].VLSKLQSPSR.[A]	[10–19]	S7: 99.72
Q9HC35	Echinoderm microtubule-associated protein-like 4	[R].ASPSPQPSSQPLQIHR.[Q]	[143–158]	S4: 99.36
Q9UDY2-7	Isoform 7 of Tight junction protein ZO-2	[R].SSEPVQHEESIRKPSPEPR.[A]	[983–1001]	S15: 99.98
Q96FF9	Sororin	[R].SGPRAPSPTKPLRR.[S]	[15–28]	S7: 99.99
Q8NE71-1	ATP-binding cassette sub-family F member 1	[K].KAEQGSEEEGEGEEEEEEGGESKADDPYAHLSK.[K]	[223–255]	S6: 100
		[R].LKKLSVPTSDEEDEVPAPKPR.[G]	[101–121]	S5: 100; T8: 100; S9: 100
Q9GZR7	ATP-dependent RNA helicase DDX24	[K].AQAVSEEEEEEEGKSSSPK.[K]	[78–96]	S5: 100
		[K].AQAVSEEEEEEEGK.[S]	[78–91]	S5: 100
		[R].KAQAVSEEEEEEEGK.[S]	[77–91]	S6: 100
		[K].VVDYSQFQESDDADEDYGRDSGPPTKK.[I]	[10–36]	S10: 100
Q9H6F5	Coiled-coil domain-containing protein 86	[R].APGSPRGQHEPSKPPPAGETVTGGFGAK.[K]	[185–212]	S4: 100
		[R].AGLGSPERPPKTSPGSPR.[L]	[54–71]	S16: 99.99
		[R].RALVEFESNPEETREPGSPPSVQR.[A]	[30–53]	S18: 99.18
Q8WWM7-3	Isoform 3 of Ataxin-2-like protein	[K].EVDGLLTSEPMGSPVSSK.[T]	[582–599]	S13: 100
		[K].STSTPTSPGPR.[T]	[678–688]	S7: 100
P52926-1	high mobility group protein HMGI-C	[R].KWPQQVVQKKPAQEETEETSSQESAEED.[−]	[82–109]	S24: 100
		[R].KWPQQVVQKKPAQEETEETSSQESAEED.[−]	[82–109]	T19: 66.67; S20: 66.67; S21: 66.67; S24: 100
		[R].KWPQQVVQKKPAQEETEETSSQESAEED.[−]	[82–109]	S20: 97.22; S21: 97.22
P19338	Nucleolin	[K].AAAAAPASEDEDDEDDEDDEDDDDDEEDDSEEEAMETTPAKGKK.[A]	[177–220]	S8: 100; S30: 100
		[K].VVVSPTKKVAVATPAK.[K]	[64–79]	S4: 99.99
P49736	DNA replication licensing factor mcm2	[R].GLLYDSDEEDEERPARK.[R]	[134–150]	S6: 100
		[R].RGLLYDSDEEDEERPARK.[R]	[133–150]	S7: 100
Q9H1E3	Nuclear ubiquitous casein and cyclin-dependent kinase substrate 1	[K].VVDYSQFQESDDADEDYGRDSGPPTK.[K]	[10–35]	S10: 100
		[R].KVVDYSQFQESDDADEDYGRDSGPPTK.[K]	[9–35]	S11: 100
		[R].LKATVTPSPVKGK.[G]	[174–186]	T6: 100; S8: 100

Peptide sequence and positions within proteins togheter with accession numbers and descriptions are reported. Localization and best site probability for phosphorylation within peptides performed with the ptmRS tool of the Proteome Discoverer are also reported.

### Functional analysis of identified phosphoproteins

**Figure 2:**
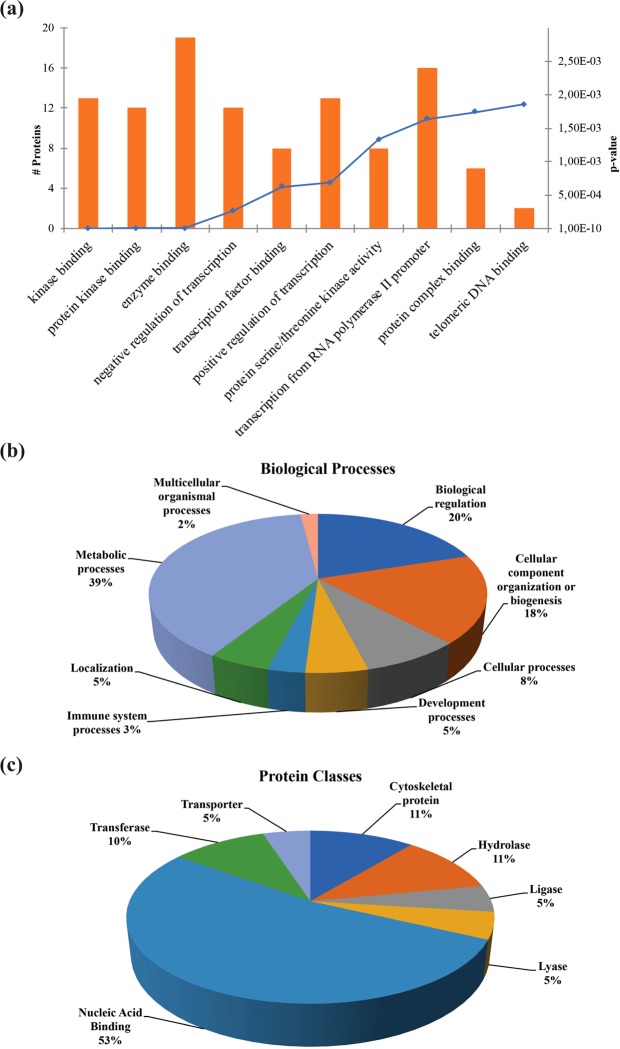
Bioinformatic analysis of WKYMVm-induced phosphoproteins. (**a**) Bar chart of the significantly enriched molecular functions of phosphoproteins uniquely identified in CaLu-6 cells following WKYMVm stimulation. The number of proteins belonging to the enriched gene ontology term and the p-values are reported on the graph. (**b**) PANTHER classification system of FPR2-dependent phosphorylated proteins in Biological Processes. (**C**) PANTHER analysis of the Protein Classes of the uniquely identified phosphoproteins.

The molecular function enrichment analysis of these proteins, performed through the Network analysis software, revealed that kinase binding, protein kinase binding, enzyme binding, proteins involved in transcription processes were among the most significantly enriched categories (Fig. 2a). We used PANTHER classification system to categorize FPR2-dependent phosphorylated proteins according to their known or postulated functions in biological processes. The analysis revealed that the phosphoproteins can be classified into eight groups and that Metabolic process represents the largest group in all the identified phosphorylated proteins (Fig. 2b). About 33% of the proteins of this group are involved in biosynthetic processes and we identified, among others, proteins involved in nucleotides, aminophosphonate, glycerophospholipid and cholesterol metabolisms, such as PRPP synthase-associated protein 2, choline-phosphate cytidylyltransferase A and hydroxymethylglutaryl-CoA synthase. The remaining 67% of proteins are involved in cellular metabolic processes, such as primary metabolism, organic substance metabolism and nitrogen compound metabolism. Biological regulation represents the second largest group in all the identified phosphorylated proteins (Fig. 2b). In particular, here we identified proteins involved in the regulation of cell cycle, cell division, apoptosis and transmembrane transport (Sororin, Promyelocytic leukemia protein PML, ATP-binding cassette). PANTHER analysis of the protein classes of the uniquely identified phosphoproteins showed that the nucleic acid binding proteins represent the largest group in all the phosphorylated proteins (Fig. 2c). About 77% of phosphoproteins of this group are RNA-binding proteins and about 23% are DNA-binding proteins. They play crucial roles in transcription, replication, recombination, DNA repair, and other nuclear functions. Notably, in this group we identified transcription and splicing regulatory proteins (RNA-binding motif protein, hRNP A3, hRNP C1 hRNP H1), proteins involved in the synthesis and maturation of ribosomes and translation initiation factors (Nucleolin, eIF-4B, eIF-4γ1), cell cycle and DNA replication regulatory proteins (Rb, MCM2), histonic and non-histonic DNA-binding proteins (Histone H1.5, HMGI-C).

**Figure 3:**
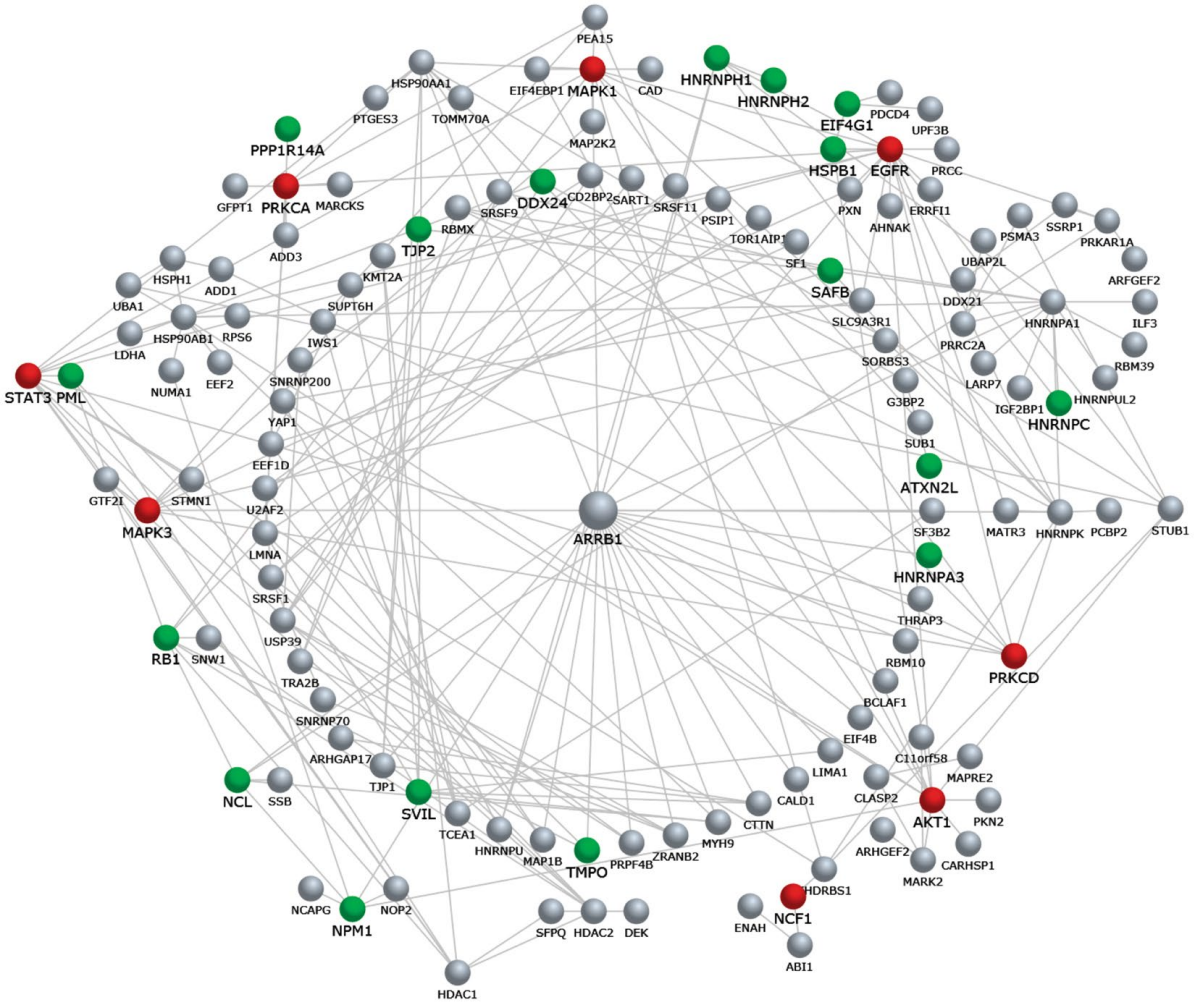
Interaction network analysis of proteins identified by LC-MS/MS. Phosphoproteins identified by LC-MS analysis are represented as grey nodes, the subset of proteins uniquely identified upon WKYMVm stimulation are indicated as green nodes, and selected phosphoproteins known to be involved in FPR2 stimulation by WKYMVm are shown as red nodes. The core molecule of the larger cluster within the network was found to converge on β-arrestin-1 (ARRB1). The network was constructed by including clusters with more than one node and excluding interactions from outside the selected dataset.

To shed light on biological relationships among identified phosphoproteins, an interaction network map was constructed in silico by using the FunRich software (Fig. 3). To this aim, the dataset of all phospho-proteins identified by MS analysis (Fig. 3, grey nodes) was integrated with selected accession numbers of proteins known to be involved in response to FPR2 stimulation by W peptide (Fig. 3, red nodes). In particular, to investigate potential connection within the network and to point out the mutual interactions of identified proteins, the following nodes were included within the experimental dataset: Erk2 (P28482), Erk1 (P27361), PKCα (P17252), PKCδ (Q05655), p47phox (P14598), EGFR (P00533), PI3K (Q8WYR1), Akt (P31749), STAT3 (P40763)^3,15,17,19^. As shown, a large subset of proteins was mapped on a cluster converging on β-arrestin-1 (ARRB1), which plays a crucial role in GPCR signaling. This cluster also includes and displays connections with several phospho-proteins uniquely identified in WKYMVm-treated cells (Fig. 3, green nodes) such as HNRPNA3, TJP2, SVIL, TMPO, RB1. Moreover, additional phosphoproteins were mapped on the network on core molecules known to be involved in FPR2 stimulation by W peptide, such as PRKCA and EGFR.

### HSP-27, MCM2, OSR1, Rb and MARCKS are selectively phosphorylated upon FPR2 stimulation by WKYMVm

FPR2 stimulation by WKYMVm exacerbates cell proliferation and the malignant phenotype of CaLu-6 cells, and its inhibition by PTX or WRWWWW significantly prevents these biological responses^16^. Therefore, we selected a subset of identified phospho-proteins (i.e. HSP-27, MCM2, OSR1, Rb and MARCKS) for further validation by western blot analysis, on the basis of their involvement in the control of cell proliferation and in signal transduction cascades triggered by FPR2 activation that we identified by MS/MS analysis.

HSP-27 (P04792) belongs to heat shock protein family and is expressed at basal level in many cell types and tissues. It is a chaperone^32^ with anti-inflammatory and immunomodulatory actions, including the release of the anti-inflammatory cytokine IL-10^33^. The protein is phosphorylated on three residues (Ser15, Ser78, and Ser82) by MAPKAP kinase 2/3/5 via the activation of p38MAPK pathway^34^. Other kinases, such as PKB, PKC, PKD, and PKG also independently phosphorylate HSP-27^35^. In fact, in SH-SY5Y human neuroblastoma cells, carbachol-dependent HSP-27 phosphorylation on Ser82 and Ser78 residues are prevented by PKC and p38MAPK inhibition, and phorbol esters-dependent HSP-27(Ser82) phosphorylation is reversed by PKC or PKD inhibitors^36^. Rac is also involved in HSP-27 phosphorylation, as demonstrated by the observation that the inhibition of Rac-guanine nucleotide exchange factor interaction reduces the collagen-induced HSP-27 phosphorylation on Ser15, Ser78 and Ser82 residues^37^.

HSP-27 phosphorylation promotes the dissociation of HSP-27 multimers into monomers and dimers that can interact with other proteins. HSP-27 phosphorylation is highly dynamics and regulates several cellular processes in a context-specific manner. Indeed, phospho-HSP-27 is known to: (i) down-regulate the expression of intracellular glutathione and, in turn, the resistance against oxidative stress^38^; (ii) prevent Fas-induced apoptosis^39^; (iii) promote actin filament polymerization^40^ and (iv) modulate estrogen signaling^41^. In addition, in a phospho-proteome analysis of tumors undergoing ischemia, HSP-27 results hyper-phosphorylated at the Ser82 residue suggesting the relevance of this site in ischemia^42^. Furthermore, in atherosclerotic lesions of hypercholesterolaemic rabbits a significant increase of HSP-27(Ser82) phosphorylation and pospho-p38MAPK was observed^43^.

**Figure 4:**
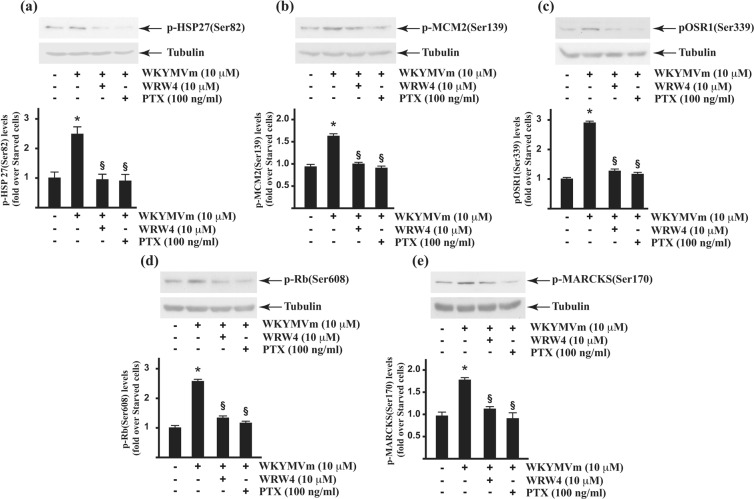
Validation of FPR2-dependent phosphoproteins. Western blot and densitometric analyses of at least three independent experiments. Fifty micrograms of whole lysates were purified from control cells and from 24 hours serum-starved CaLu-6 cells stimulated with 10 μM WKYMVm for 5 minutes, in the presence or absence of 10 μM WRWWWW (WRW4) or 100 ng/mL PTX. Lysates were resolved on 10% SDS-PAGE and hybridized with anti phospho-selective antibodies specific for p-HSP-27(Ser82) (**A**); p-MCM2(Ser139) (**b**); p-OSR1(Ser339) (**c**); p-Rb(Ser608) (**d**) and p-MARCKS(Ser170) (**e**). An anti-tubulin antibody was used as loading proteins control.

Consistent with previous evidences reporting the induction of p38MAPK activity by WKYMVm^3,44^, our MS/MS analysis revealed FPR2-dependent phosphorylation of HSP-27 on Ser82 residue (Table 1). Accordingly, western blot experiments performed with an anti-posphoHSP-27(Ser82) antibody showed an increased phosphorylation of HSP-27 in WKYMVm-stimulated cells, which is prevented by cells treatments with the FPR2 antagonist WRW4 or with PTX, which catalyzes the ADP-ribosylation of the αi subunits of the heterotrimeric G proteins. (Fig. 4a).

Stimulation of human monocytic U937 cells, or primary monocytes, or FPR2-transfected HEK293 cells with ANXA1 induces downstream phosphorylation of p38MAPK, MAPKAP kinase, and HSP-27. Pro-inflammatory FPR2 agonists, such as LL-37 and Serum-Amyloid Alpha (SAA) are unable to phosphorylate HSP-27, suggesting that the activation of p38MAPK/MAPKAPK/HSP-27 pathway is a response triggered by pro-resolving, and not by pro-inflammatory ligands, upon FPR2 activation^45^. Interestingly, FPR2 stimulation of monocytes with ANXA1 induces the release of IL-10, which is prevented by pretreatment with a p38MAPK inhibitor^45^. Other members of the FPR family induce HSP-27 activation. In the human colonic epithelial cell line Caco2, physiological concentrations of N-fMLP significantly increase HSP-27 expression which is prevented by the FPR1 receptor antagonist BOC-FLFLF. In these cells N-fMLP activates p38MAPK and ERK1/2, but only p38MAPK inhibition prevents N-fMLP-dependent induction of HSP-27^46^.

MCM2 (P49736) belongs to the protein family of “ATPases associated with diverse cellular activities” (AAA+). It is a component of the MCM2-7 complex, which is the putative replicative helicase necessary for DNA replication initiation and elongation in eukaryotic cells. MCM2 can be phosphorylated by different kinases and these phosphorylation events are involved in DNA replication, cell cycle progression and checkpoints regulation. Cdc7/Dbf4 phosphorylates MCM2 and other MCMs^47^ both *in vivo* and *in vitro*, on Ser27, Ser41, and Ser139 residues^48^, whereas Cdks phosphorylate MCM2 but not on Ser139 residues^47^. In human cells, Cdc7 is activated by its regulatory subunits Dbf4 and Drf1^49,50^ and Cdc7/Dbf4 complex is directly involved in the initiation of DNA replication by targeting MCM2^48^. Casein kinase 2 (CK2) and salt-inducible kinase 1 (SIK1) also phosphorylate MCM2 on Ser139 *in vitro*, but there is not evidence that CK2 is responsible for this phosphorylation *in vivo*^51,52^. EGFR- and ERKs-dependent activation of CK2 phosphorylates phosphoglycerate kinase 1 (PGK1), resulting in PGK1/Cdc7 interaction. Cdc7-bound PGK1 converts the ADP in ATP thus removing ADP inhibition on Cdc7 and promoting MCM2 phosphorylation on Ser139^53^. Previously, we demonstrated that WKYMVm stimulation of CaLu-6 cells induces EGFR transactivation and ERKs phosphorylation^16^, and accordingly to EGFR-dependent activation of CK2/PGK1/Cdc/7 cascade, our results potentially explain the observed FPR2-dependent phosphorylation of MCM2 on the Ser139 residue (Table 1). Western blot experiments performed with an anti-pMCM2(Ser139) antibody showed an increased phosphorylation levels of MCM2(Ser139) in FPR2-stimulated cells, and preincubation of CaLu-6 cells with WRW4 or PTX before W peptide stimulation prevent this phosphorylation (Fig. 4b).

The regulatory role of MCM2 in lung cancer has been extensively investigated in a integrate analysis of phospho-proteome and proteome of overexpressed and silenced MCM2 lung cancer cells^54^. Such analysis demonstrated a phosphoMCM2-regulated functional network, suggesting that the deregulation of MCM2 phosphorylation is involved in lung cancer cell proliferation, cell cycle, and migration and that potential target cancer-specific phospho-proteins can be identified by the analysis of molecular interactions of phosphorylated MCM2^54^. The role of phosphorylated MCM2 in cancer is also corroborated by a phospho-proteomic analysis of liver cell lines with different proliferation potential. The results show that MCM2 is hyper-phosphorylated in liver cancer in particular on a novel Thr27 phosphosite, but also on Ser139 residue^55^. In these cells, MCM2 promotes cell proliferation via the regulation of high mobility group protein HMG-I/HMG-Y (HMGA1) phosphorylation^55^.

The oxidative stress–responsive kinase 1 OSR1 (O95747) is a serine/threonine-protein kinase involved in the regulation of the solute carrier 12 family of cation-chloride cotransporters and thereby in the modulation of cellular ion homeostasis, blood pressure, hearing, and kidney functions^56,57^. OSR1 is activated by “with no lysine” (WNK) protein kinase family, which phosphorylates a Thr185 residue in the T-loop kinase domain, and Ser325 and Ser339 residues in the S-domain of OSR1. The role of OSR1(Ser325) and OSR1(Ser339) phosphorylations is unclear^58^. Some evidence suggests that since the S-domain of OSR1 contains a WEW motif (aminoacids 336–338), essential for binding to the scaffolding protein MO25, the phosphorylation on serine residues adjacent to WEW motif (Ser339) could enhance binding to MO25^58^. PI3K-Akt signaling activates the WNK-OSR1 cascade^59^ by Akt-dependent phosphorylation of WNK on Thr60, which is prevented by PI3K inhibitors^60^. WNK3 is a direct target of Akt^61^ and is subjected to phosphorylation triggered by EGF-dependent PI3K-Akt pathway^59^. Akt activity is regulated not only by PI3K phosphorylation in the activation loop (Thr308) but also by mammalian target of rapamycin complex 2 (mTORC2) phosphorylation in C-terminal hydrophobic motif (Ser473)^57^. mTORC2 also phosphorylates OSR1 on Ser339 residue, increasing OSR1 activity^62^, and inhibition of mTORC2 does not prevent WNK activity, indicating that mTORC2 regulates OSR1 independently by WNK^57^. Accordingly, OSR1(Ser339) phosphorylation has been identified by MS in phospho-proteomic studies to define the signaling networks downstream of mTORC1 and mTORC2^63,64^. A phospho-proteomics analysis of hydrogen peroxide-induced fibroblasts derived from normal individuals and A-T patients suggests that also ROS play a role in OSR1 phosphorylation^65^. OSR1 phosphorylation on Ser339 residue observed in WKYMVm-stimulated CaLu-6 cells (Table 1), suggests a link between FPR2-PI3K-Akt-^3,13,19^ and Akt-mTORC2-OSR1 cascades. Consequently, western blot analysis performed with an anti-pOSR1(Ser339) antibody shows an increase of OSR1(Ser339) phosphorylation in FPR2-stimulated cells, which is prevented by WRW4 or PTX (Fig. 4c).

The retinoblastoma tumor suppressor protein, Rb (P06400), negatively regulates entry into S phase. The binding of Rb to E2F prevents the release of E2F, thereby inhibiting the entry of the cell cycle. Therefore, Rb acts as a cell cycle inhibitor and is activated by multisite Cdk-dependent phosphorylation in response to positive growth signals^66,67^. The Rb protein consists of several domains (RbN, RbIDL, RbC), as well as a large loop within the pocket domain (RbPL). Cdk-dependent Rb phosphorylation occurs at 13 consensus sites which include all these regions^67^. Unphosphorylated Rb on Ser608 residue has growth suppressive activity and specifically contact the COOH-terminus of MCM4. Phosphorylation of Ser608 in RbPL is sufficient for partial inhibition of E2F binding^66^, whereas Ser608/Thr373 phosphorylation produces unique conformational changes of the Rb structure that result in the allosteric and complete inhibition of the E2F binding^67^. Ser608/Ser612 phosphorylation induces an intramolecular association between RbPL and the pocket domain. This association obstructs the E2FTD binding site in the pocket, preventing the simultaneous binding of phosphorylated RbPL and E2FTD^67^.

Rb is not phosphorylated on Ser608 residue and is tethered to nuclear structures in CD34+ hemopoietic progenitor cells, suggesting that it has growth-suppressive activity, whereas Rb(Ser608) is phosphorylated by CDK4/6 complexes in acute lymphoblastic leukemia cells^68^. The role of Rb(Ser608) phosphorylation in the control of the progression of the cell cycle is further confirmed in a subset of tumor-derived cell lines, where conversion of unphosphorylated Rb in a phosphorylated form does not arise^69^. In these defective cells, a lack of dephosphorylation of Rb(Ser608) is observed. Several members of PP1 phosphatase family are implicated in the dephosphorylation, but there is no evidence for defective or deregulated expression of catalytic PP1 cores^69^. We decided to validate the identified Rb(Ser608) phosphorylation on the basis of its role in the control of cell cycle and, in western blot analysis, we observed that an anti-pRb(Ser608) antibody confirmed this phosphorylation in FPR2-stimulated cells, which was prevented by preincubation with WRW4 or PTX (Fig. 4d).

MARCKS (P29966) is a membrane-binding protein and is the main substrate of PKC in many cell types. It binds calmodulin (CaM), actin, synapsin and is involved in cell migration, regulation of cell cycle, actin-filament reorganization, adhesion, cytoskeletal control and neurosecretion^70^. MARCKS consists of three conserved regions, which include the phosphorylation site domain (PSD), also called effector domain (ED), which is necessary for its association to PIP2. PKC-dependent phosphorylation on Ser159, Ser163, and Ser170 residues within PSD increases MARCKS detachment from the membrane and removes PIP2 association^71^.

PKC-dependent phosphorylation of MARCKS reduces its affinity for CaM, suggesting that MARCKS could represent the storage of CaM and that phospho-MARCKS could play a crucial role in regulating CaM availability within the cell^70^. MARCKS is preferentially phosphorylated by PKCα but also by PKCδ and PKCε, which phosphorylate Ser159, Ser163, and Ser170 residues in the PSD of human MARCKS^72,73^. These phosphorylations mask positive charges on the protein which, in turn, segregates from the plasma membrane to the cytoplasm. Rho kinase (ROCK) also phosphorylates PSD of MARCKS^74^ and, in mouse bone granulocytes, W peptide stimulation triggers ROCK activation^75^. We previously observed the ability of FPR2 to activate PKCα and PKCδ in several cell types^3,17,19^ and, accordingly, our phospho-proteomic analysis identified the FPR2-dependent MARCKS(Ser170) phosphorylation (Table 1). In protein extracts from WKYMVm-stimulated cells, the selective phosphorylation of MARCKS(Ser170) was detected by a specific antibody and this phosphorylation is prevented by the FPR2 antagonist and by Gi protein inhibition (Fig. 4e). These results are in line with previous evidence reporting that stimulation of FPR1 with its agonist N-fMLP triggers p47^phox^ and MARCKS phosphorylation^76^. In the lung, phospho-MARCKS plays a key role in the control of mucin secretion and inflammation. Higher phospho-MARCKS levels are correlated with shorter overall survival of lung cancer patients, suggesting a key role for MARCKS in lung cancer and a therapeutic strategy for inhibiting PKC activity^77^.

**Figure 5:**
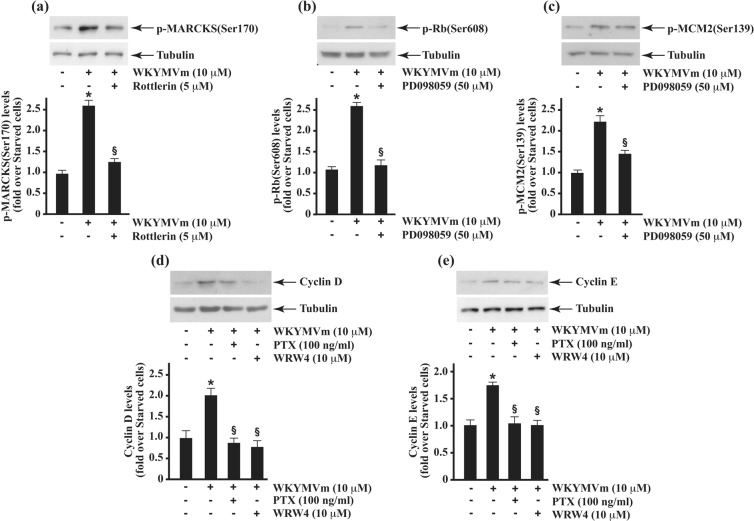
A MEK and a PKC inhibitor prevent Rb(Ser608), MCM2(Ser139) and MARCKS(Ser170) phosphorylation. Fifty micrograms of proteins were purified from control cells and from 24 hours growth-arrested cells stimulated with 10 μM W peptide for 5 minutes, in the presence or absence of 50 μM PD098059 (Panel a,b) or 5 μM Rottlerin (Panel c). Lysates were resolved on 10% SDS-PAGE and hybridized with anti phospho-selective antibodies specific for p-Rb(Ser608) (**a**); p-MCM2(Ser139) (**b**); and p-MARCKS(Ser170) (**c**). An anti-tubulin antibody was used as loading proteins control. Densitometric analyses was performed on at least three independent experiments.

We also used specific inhibitors to determine the interconnection between some of the signalling readouts identified. PKC and MAPK represent two nodes in the interaction network analysis of proteins identified by LC-MS/MS (Fig. 3). Therefore, we preincubated cells with Rottlerin or PD098059 prior WKYMVm stimulation, and in western blot experiments we observed that the inhibition of PKCδ, which is activated upon FPR2 stimulation^3,17,19^, prevents WKYMVm-induced MARCKS(Ser170) phosphorylation (Fig. 5a). Moreover, the MEK inhibitor prevents both Rb(Ser608) and MCM2(Ser139) phosphorylation (Fig. 5b,c). We evaluated functional consequences of Rb and MCM2 phosphorylation, and we observed an increase of Cyclin D and Cyclin E expression in FPR2-stimulated cells (Fig. 5d,e). These results strengthens the notion on the ability of FPR2 to trigger Ras/MAPK cascade, mitogenic stimuli and, in turn, cell cycle progression.

### FPR2 stimulation with anti-inflammatory agonists induces HSP-27, OSR1, Rb and MARCKS phosphorylation

**Figure 6:**
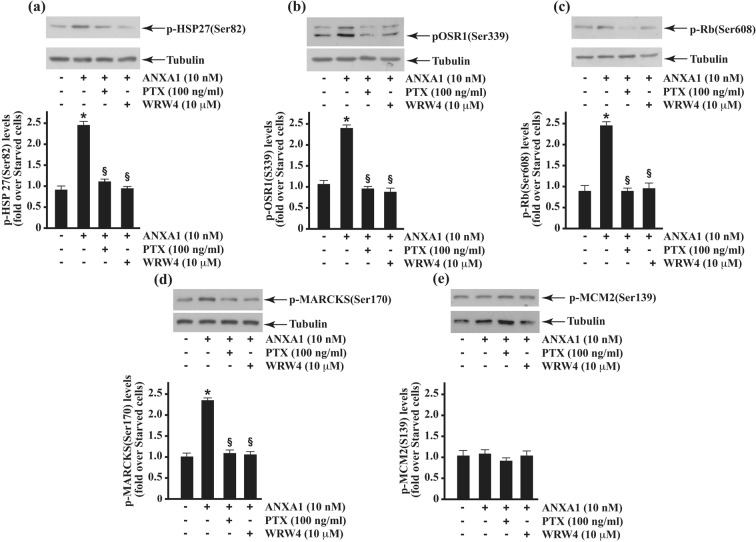
HSP-27, OSR1, Rb and MARCKS phosphorylation depends on ANXA1 stimulation. Serum-starved CaLu-6 cells were stimulated or not with 10 nM ANXA1 for 5 minutes, in the presence or absence of 10 μM WRW4 or 100 ng/mL PTX. Fifty micrograms of whole lysates were purified, resolved on 10% SDS-PAGE and hybridized with anti-p-HSP-27(Ser82) (**a**); anti-p-OSR1(Ser339) (**b**); anti-p-Rb(Ser608) (**c**); anti-p-MARCKS(Ser170) (**d**), and anti-p-MCM2(Ser139) antibodies. Proteins were detected by autoradiography and bands densitometry was estimated using a Discover Pharmacia scanner. The filters were reprobed with an anti-tubulin antibody to normalize the amount of loaded proteins. Data are representative of three independent experiments.

**Figure 7:**
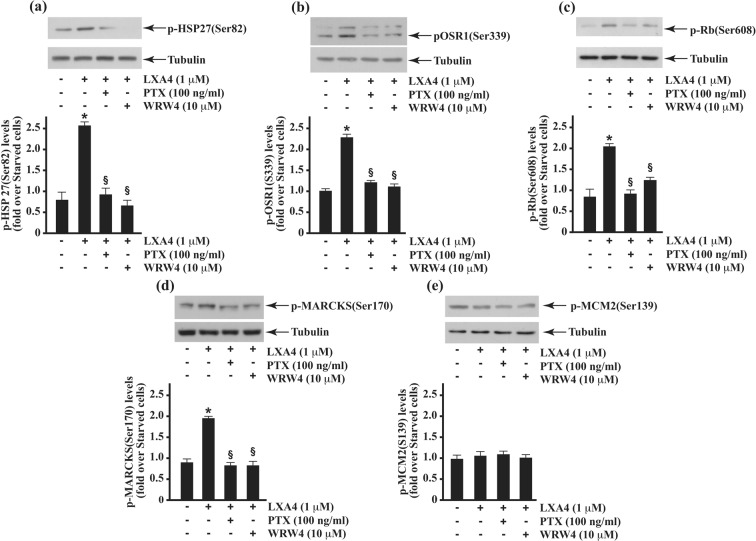
LXA4 induces HSP-27, OSR1, Rb and MARCKS phosphorylation. CaLu-6 cells were serum-starved for 24 hours and stimulated or not with 1 μM LXA4 for 5 minutes. Cells were also preincubated with 10 μM WRW4 or 100 ng/mL PTX before LXA4 stimulation. Whole lysates (50 μg) were resolved on 10% SDS-PAGE and hybridized with anti-p-HSP-27(Ser82) (**a**); anti-p-OSR1(Ser339) (**b**); anti-p-Rb(Ser608) (**c**); anti-p-MARCKS(Ser170) (**d**), and anti-p-MCM2(Ser139) antibodies. Proteins were detected by autoradiography and bands were estimated from densitometric scanning. An anti-tubulin antibody was used to normalize the amount of loaded proteins. Results are representative of three independent experiments performed in triplicate.

Several data support the development of WKYMVm as a novel and effective anti-inflammatory therapeutic agent. It shows pleiotropic, immunomodulatory, anti-apoptotic, and anti-inflammatory effects in several pathological conditions^78^, inhibits the production of pro-inflammatory cytokines (TNF-α, IL-1β, and IL-6) and enhances the levels of anti-inflammatory cytokines (IL-10 and TGFβ)^79^. FPR2 can to transmit both pro- and anti-inflammatory signals and this duality was originally established by the nature of the ligands. In general, short and flexible structures, like that of formylated peptides, activate a pro-inflammatory cell response, while ANXA1 and LXA4 are well-known anti-inflammatory FPR2 ligands. The shift between pro- and anti-inflammatory cell responses is caused by conformational modifications of FPR2 upon ligand binding, but are not merely due to ligand-receptor interaction. FPR2 can dimerize and this conformational change could account for its biological functions. Anti-inflammatory agonists enhance the formation of FPR2 homodimers and the release of inflammation-resolving cytokines, while inflammatory ligands, such as SAA, do not cause receptor homodimerization. Heterodimers of FPR2 with other members of the FPR family can transduce proapoptotic signals^43^. We stimulated CaLu-6 cells with two anti-inflammatory agonists and, in western blot experiments we observed that ANXA1 and LXA4 induce HSP-27(Ser82), OSR1(Ser339), Rb(Ser608), and MARCKS(Ser170) phosphorylation, which was prevented by PTX and WRW4 (Figs. 6A–D and 7A–D). However, no significant variation was detected in MCM2(Ser139) phosphorylation (Figs. 6E and 7E), suggesting that the latter only depend on WKYMVm-stimulation.

ANXA1 stimulation of FPR2-expressing cells induces the phosphorylation of p38MAPK/MAPKAP/HSP-27 cascade^43,80^, but in these cells, the phosphorylation of HSP-27 on a Ser82 residue has not been demonstrated. Anti-inflammatory ligands increase the formation of FPR2 homodimers and/or FPR1/FPR2 heterodimers and, in turn, the release of anti-inflammatory cytokines^43^. Since CaLu-6 cells don’t express FPR1^16^, we can exclude FPR1/FPR2, but not FPR2/FPR3 heterodimerization.

## Conclusions

FPR2 was initially detected in phagocytic leukocytes, but the receptor was subsequently identified in other cell types and tissues, where it is involved in many physio-pathological processes and where it shows further functions than those normally exerted in polymorphonuclear cells. For instance, FPR2 promotes the malignancy of colon cancer^81^, triggers c-Met and EGFR transactivation in PNT1A and CaLu-6 cells^16,19^, respectively, and elicits NOX2-dependent superoxide generation in IMR90 human fibroblasts^17^, suggesting a specific role of FPR2 in non-phagocytic cells. CaLu-6 is a human lung anaplastic cancer cell line which expresses high levels of EGFR as well as a biological functional FPR2. We already demonstrated that stimulation with W peptide exacerbates the malignant phenotype of these cells through a molecular mechanism which involves EGFR tyrosine trans-phosphorylation, p47^phox^ phosphorylation, NADPH-oxidase-dependent superoxide generation, and c-Src kinase and STAT3 activity^16^. FPR2 inhibition significantly prevents cell proliferation and the malignant phenotype, suggesting the potential role of FPR2 signalling and, in turn, of phosphorylated proteins as drug targets in lung cancer epithelial cells^16^.

The investigation of the complex and dynamic regulation systems of protein phosphorylation represents an attractive field of proteomic research. The outline of the cellular phosphorylation status is crucial for the identification of intracellular signalling pathways and, in turn, for the understanding of the molecular mechanisms responsible for cancer and other human disorders. Here we report the first phospho-proteomic analysis of FPR2-stimulated CaLu-6 cells that allowed the overall identification of 290 phosphorylated proteins; among them, we found 53 unique phosphopeptides that mapped on 40 proteins with crucial roles in metabolic processes and biological regulation. In particular, we identified FPR2-induced phosphorylated proteins involved in the control of cell cycle progression, cell division and apoptosis, and thereby implicated in molecular mechanisms of cellular invasion and metastasis. Interestingly, most of these phospho-proteins are transcriptional factors or are regulators of replication, recombination, transcription, splicing, translation processes and DNA repair. The heterogeneity and complexity of phospho-protein patterns are indicative of the activity of multiple signal transduction pathways triggered by FPR2 stimulation. Several novel phosphosites have been identified in this study, which represent an important basis for further functional studies that may contribute to the identification of new signaling networks. However we cannot exclude that the phosphorylation pattern that we observed is a general phenomenon that occurs during FPR2 activation in all FPR2-expressing cells, rather than specific of lung epithelial carcinoma CaLu-6 cells.

FPR2 can transmit both pro- and anti-inflammatory signals and this ability is determined by the nature of the ligands and by the formation of higher-order structures. For instance, FPR2 can to homodimerize or to heterodimerize with FPR1 or FPR3^43^. Pro-resolving ligands, such as ANXA1 and Ac2-26, increase the formation of FPR2 homodimers and/or FPR1/FPR2 heterodimers, which led to the release of inflammation-resolving cytokines, while pro-inflammatory agonists do not cause homodimerization^43^. The receptor oligomerization is not limited to the FPR family members but also includes the scavenger receptor MARCO and is connected to agonist-evoked modifications in ERKs phosphorylation and cAMP levels^82^. We can exclude FPR1/FPR2 heterodimerization in CaLu-6 cells since these cells don’t express FPR1, but we cannot rule out the FPR2/FPR3 heterodimerization. Our results strongly suggest a potential FPR2 homodimerization upon WKYMVm stimulation, since FPR2 oligomer contributes to the activation of the p38/MAPKAPK/HSP-27 pathway^43,80^ and pro-resolving ligands, such as ANXA1, which increases the formation of FPR2 homodimers, induces the phosphorylation of HSP-27 and of other validated proteins (Fig. 6).

The identification of the intracellular signalling pathways triggered by FPR2, as well as of downstream phosphorylated proteins, will provide a better understanding of the role of FPR2 in non-phagocytic cells in physiological and pathological conditions. Furthermore, these results could provide new insights into FPR2 signalling as possible drug targets in human diseases, including inflammatory disorders, infections, and cancer, that involve this receptor.

## Supplementary information

Supplementary Table 1

Supplementary Table 2
